# Triple Tendon Transfer of the Levator Scapulae, Rhomboid Major, and Rhomboid Minor to Reconstruct the Paralyzed Trapezius

**DOI:** 10.1016/j.eats.2022.08.028

**Published:** 2022-11-17

**Authors:** Victor Hoang, Joseph Meter, Taylor Anthony, Ajit Amesur, Bassem T. Elhassan

**Affiliations:** a Brigham and Women’s Hospital, Harvard Medical School, Boston, Massachusetts, USA; b Valley Hospital Medical Center, Las Vegas, Nevada, USA; c Touro University Nevada College of Osteopathic Medicine, Henderson, Nevada, USA; d Massachusetts General Hospital, Boston, Massachusetts, USA

## Abstract

Trapezius paralysis is a relatively uncommon condition that orthopaedic surgeons may encounter. Despite the paucity, it presents as a debilitating condition with sequelae of poor function and deconditioning. Conservative management often fails, and patients are left with limited surgical options. In the current Eden-Lange procedure, tendon transfer of the levator scapulae, rhomboid major, and rhomboid minor is performed to reconstruct the paralyzed trapezius. Although good outcomes have been found with this technique, the pull of the levator scapulae and the pull of the rhomboids are in opposition to each other, which presents a biomechanical problem for patients because this fails to re-create the natural function of the trapezius. In this article, we present a technique that is a modification of the Eden-Lange triple tendon transfer using suture bone bridges in which the levator scapulae is transferred as with the original procedure; however, the rhomboids with bony bridges are transferred to a different point along the medial scapula. Our technique therefore may better re-create the natural pull of the fibers of the upper, middle, and lower trapezius.

Trapezius paralysis is a relatively rare condition most associated with iatrogenic spinal accessory nerve damage from a lymph node biopsy procedure, radical neck dissection, and blunt or penetrating trauma to the posterior cervical triangle.^1^ The trapezius muscle functions to rotate the scapula and assist in shoulder abduction. Trapezius paralysis in turn can result in shoulder drooping, pain, weakness, loss of shoulder abduction, and lateral scapular winging.^2^ Conservative management including transcutaneous nerve stimulation, nonsteroidal anti-inflammatory drugs, scapular bracing, and physical therapy historically has shown a poor prognosis, and surgical intervention in the form of an Eden-Lange triple tendon transfer after conservative treatment failure is often ultimately recommended.^3^ The Eden-Lange technique involves transfer of the levator scapulae (LS) to the lateral scapular spine, along with transfer of the rhomboid minor (Rm) and rhomboid major (RM) to the infraspinatus fossa, to restore trapezius function. Recently, a modified Eden-Lange technique has been described, involving Rm and RM transfer to the scapular spine just medial to the LS transfer.^2^ In this article, we describe the operative steps of the modified Eden-Lange triple tendon transfer for trapezius paralysis.

## Surgical Technique

### Indications

This technique is indicated for patients with trapezius palsy in whom conservative management has failed, including physical therapy, nerve stimulation, nonsteroidal anti-inflammatory drugs, and scapular bracing.

### Materials

Our technique uses the following items: 7-mm drill bit, Stryker power drill (Kalamazoo, NJ), 10 double sutures with No. 2 OrthoCord (DePuy Orthopaedics, Warsaw, IN), suture passer, 14- or 16-gauge Angiocath (Medline, Northfield, IL), straight needle, needle driver, suture scissors, vancomycin powder, and Hemovac drain (Zimmer Biomet, Warsaw, IN).

### Patient Position

The patient is placed in the semi-lateral decubitus position with bony prominences and subcutaneous nerve sites well padded. The operative site of the scapular region including the ipsilateral upper extremity is prepared and draped in sterile fashion.

### Surgical Approach

**Fig 1:**
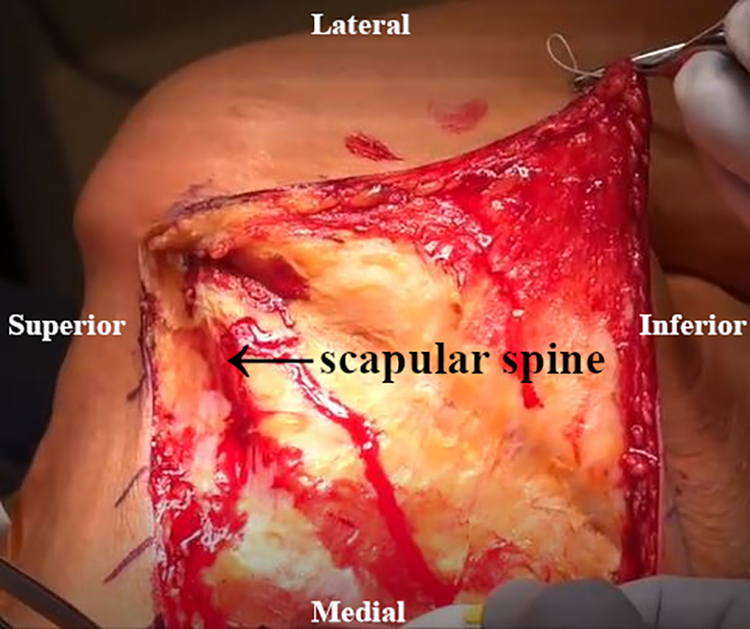
Intraoperative image of right scapula from posterior viewpoint. An inverted L–shaped incision is made proximal and medial to the tip of the scapula. This is then extended proximal to the medial scapular spine and curved laterally toward the medial aspect of the mid acromion.

**Fig 2:**
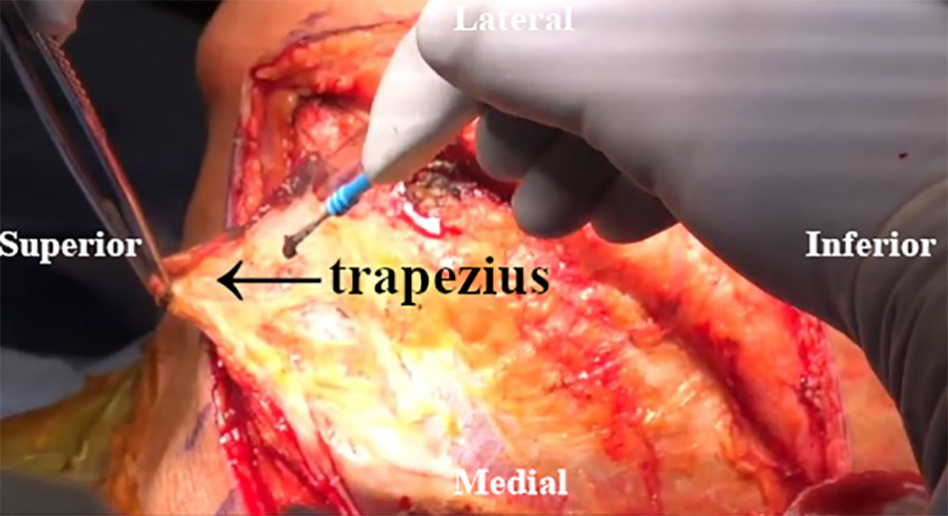
Intraoperative image of right scapula from posterior viewpoint. The paralyzed trapezius muscle insertions are detached and reflected from the scapula and acromion.

**Fig 3:**
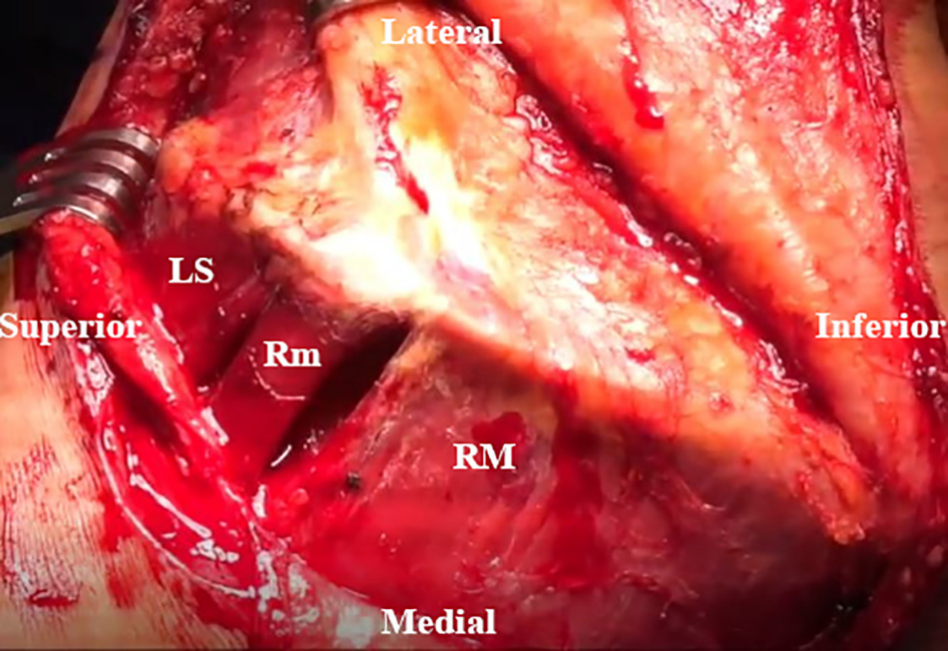
Intraoperative image of right scapula from posterior viewpoint. The levator scapulae (LS), rhomboid minor (Rm), and rhomboid major (RM) are exposed by pulling the scapula distally, and intervals are developed.

**Fig 4:**
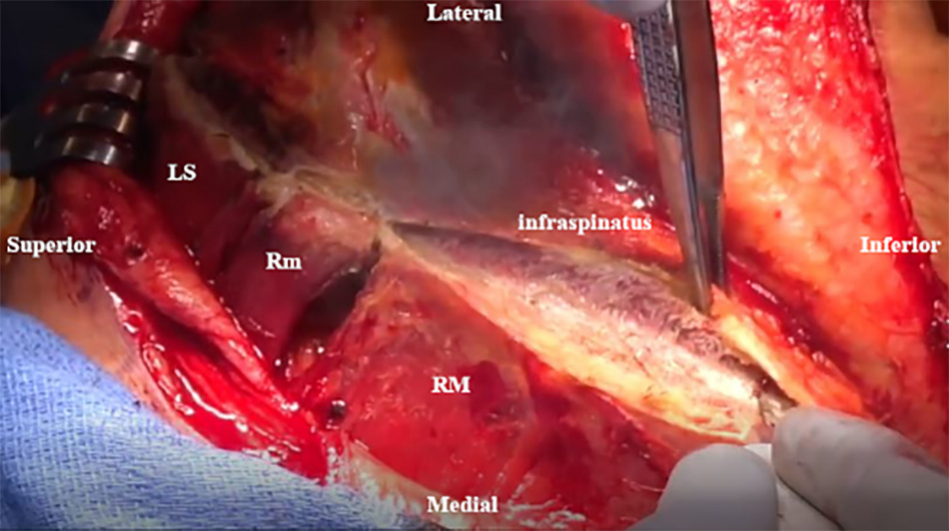
Intraoperative image of right scapula from posterior viewpoint. The supraspinatus and infraspinatus origins are minimally elevated for greater exposure of the levator scapulae (LS), rhomboid minor (Rm), and rhomboid major (RM) bony insertions.

An inverted L–shaped incision is made starting 1 cm proximal and 2 cm medial to the tip of the scapula (Video 1). The incision is extended proximal to the medial spine of the scapula and curved laterally toward the medial aspect of the mid acromion (Fig 1). Skin flaps are created and reflected proximally and distally. The paralyzed trapezius is dissected and reflected medially as a flap. The insertions of the lower trapezius muscle, middle trapezius muscle, and most of the upper trapezius muscle are detached and reflected from the scapula and acromion (Fig 2). The patient’s hand is placed behind the back to allow scapular winging from the chest wall. Exposure of the LS, Rm, and RM is achieved by traction on the scapula distally. Muscular intervals are then developed, with careful attention to avoid injury to the underlying dorsal scapular nerve (Fig 3). Minimal elevation of the supraspinatus origin and infraspinatus is performed to expose the bony insertions of the LS, Rm, and RM (Fig 4).

### Tendon Detachment and Transfer Preparation

**Fig 5:**
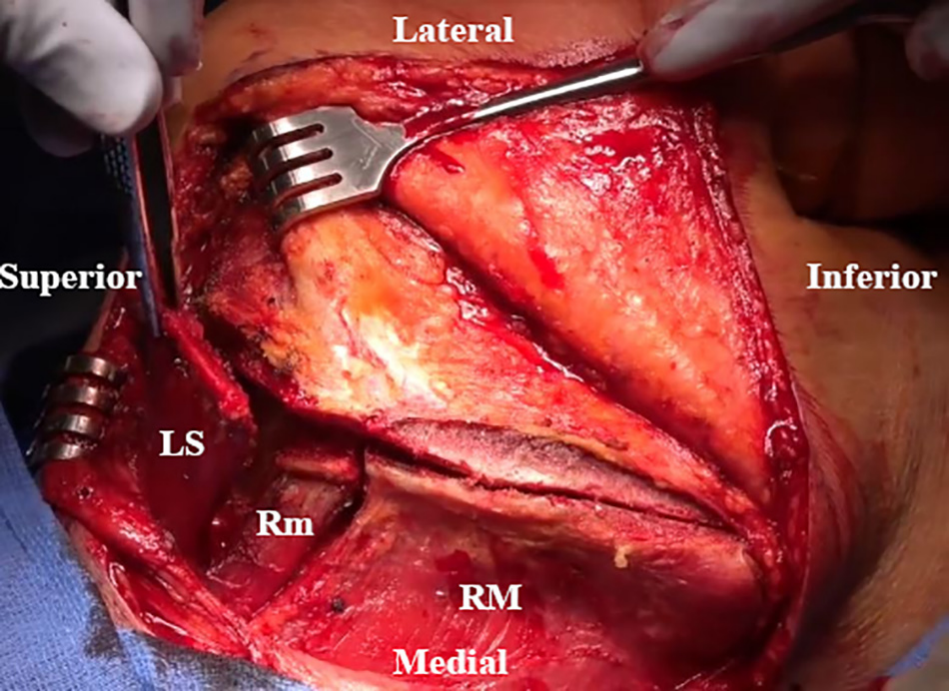
Intraoperative image of right scapula from posterior viewpoint. An oscillating saw is used to create osteotomies of the levator scapulae (LS), rhomboid minor (Rm), and rhomboid major (RM) insertions.

**Fig 6:**
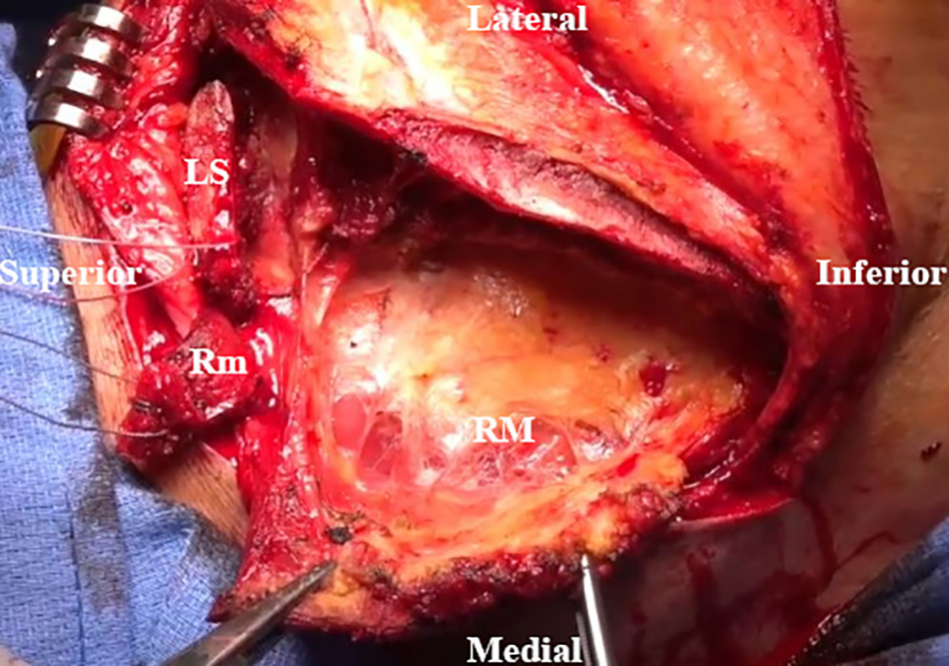
Intraoperative image of right scapula from posterior viewpoint. The detached muscles are tagged with sutures and pulled medially. The undersurface is subsequently dissected to identify and protect the dorsal scapular nerve. (LS, levator scapulae; Rm, rhomboid minor; RM, rhomboid major.)

**Fig 7:**
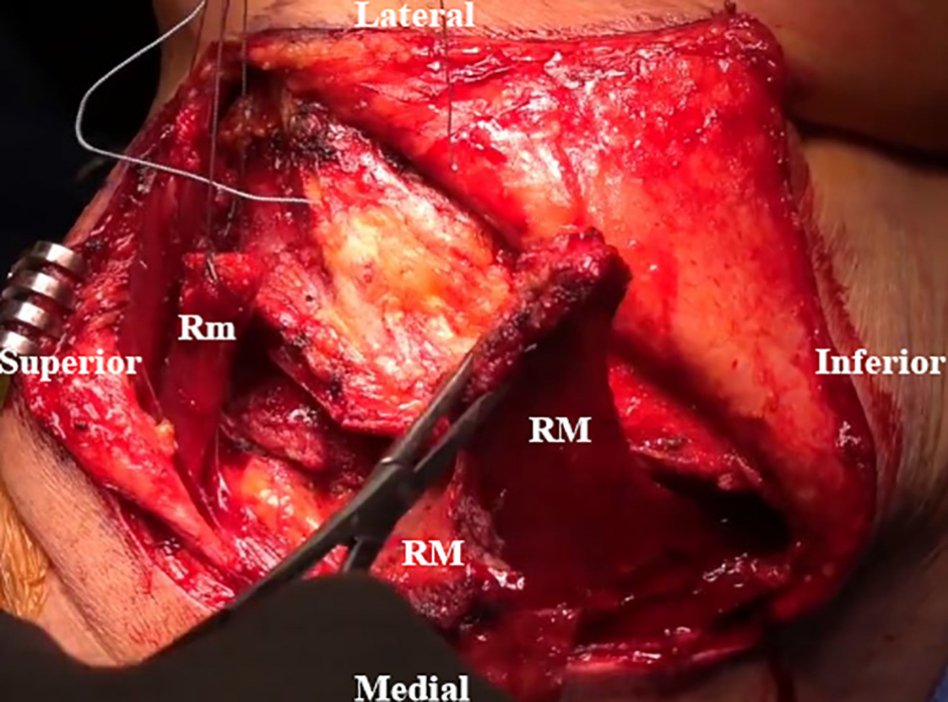
Intraoperative image of right scapula from posterior viewpoint. A transverse incision of the bony attachment of the rhomboid major (RM) is made to separate it into 2 parts. (Rm, rhomboid minor.)

**Fig 8:**
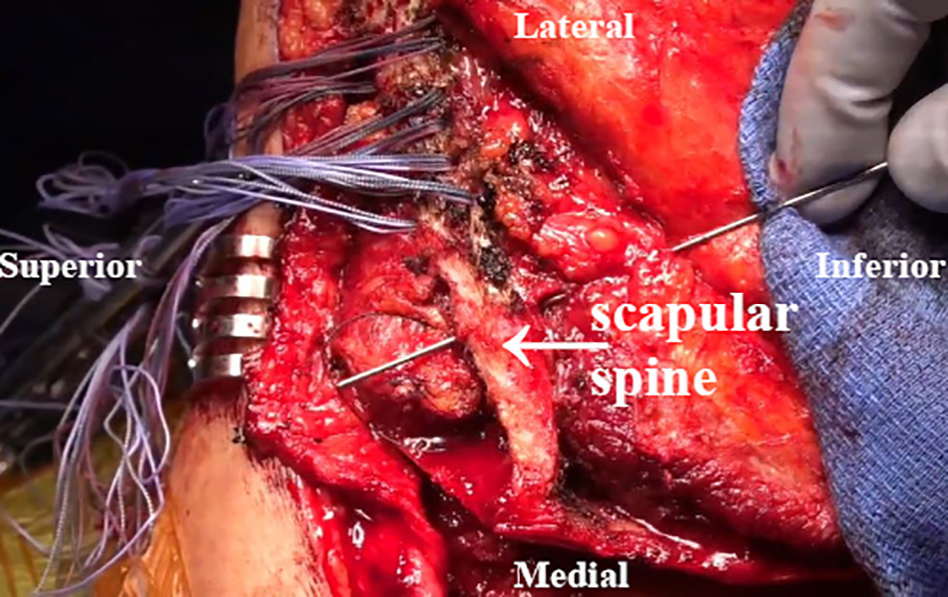
Intraoperative image of right scapula from posterior viewpoint. Transverse osseous tunnels are created using electrical wire and double suture with No. 2 OrthoCord.

An osteotomy of the LS, Rm, and RM insertions is performed with an oscillating saw. The scapular spine and acromion are then exposed (Fig 5). Tag sutures are placed through the detached muscles, which are retracted medially, with careful dissection of the undersurface to avoid injury to the dorsal scapular nerve (Fig 6). A transverse incision is made to cut the RM’s bony attachment into 2 parts owing to the prominent length (Fig 7). The surface of the spine of the scapula and medial posterior aspect of the acromion is then debrided to a bleeding base. Transverse osseous tunnels are consecutively drilled and fixation is begun with electrical wire and double suture with No. 2 OrthoCord as follows: 5 tunnels for the LS, 1 for the Rm, and 4 for the RM (Fig 8).

### Tendon Transfer

**Fig 9:**
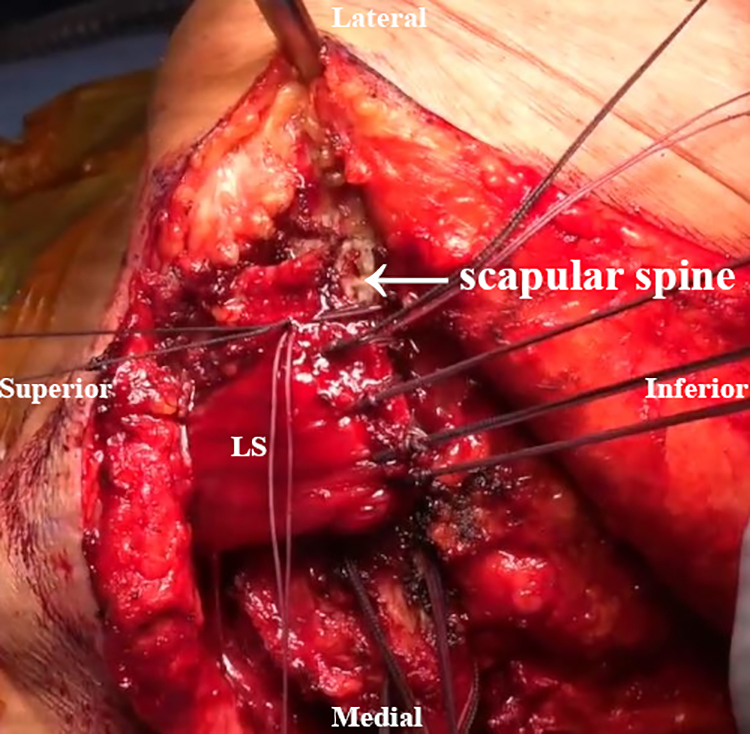
Intraoperative image of right scapula from posterior viewpoint. The levator scapulae (LS) is transferred to the scapular spine slightly posterior to the posterior aspect of the acromion.

**Fig 10:**
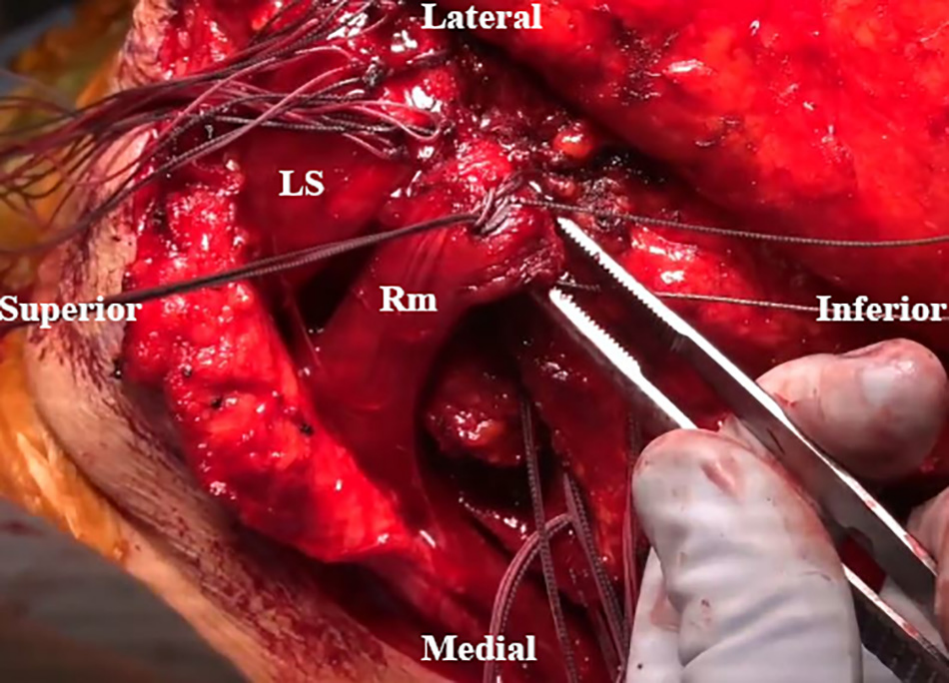
Intraoperative image of right scapula from posterior viewpoint. The rhomboid minor (Rm) is transferred slightly medial to the previously transferred levator scapulae (LS) along the scapular spine.

**Fig 11:**
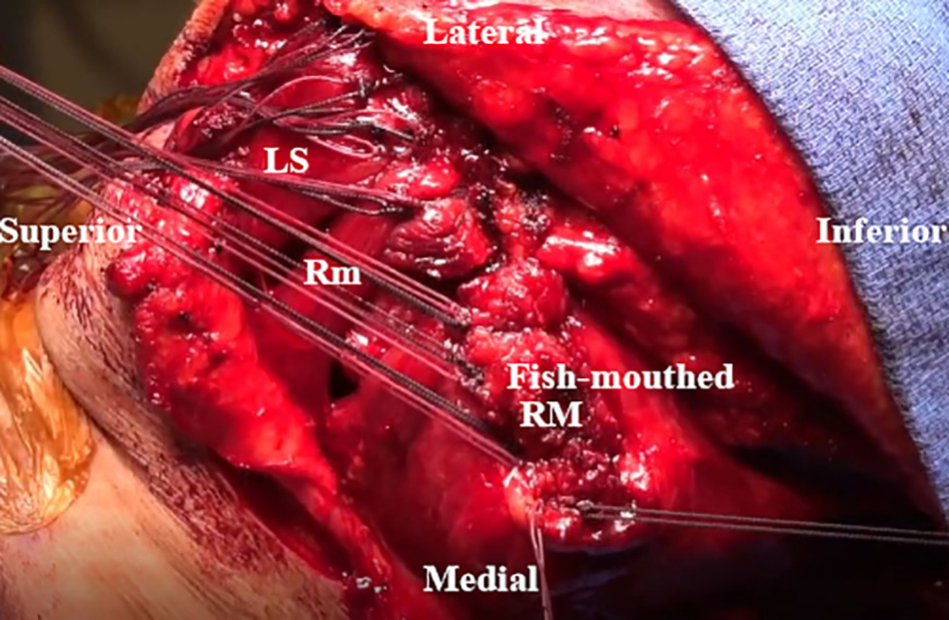
Intraoperative image of right scapula from posterior viewpoint. The split bony attachment of the rhomboid major (RM) is fish-mouthed and attached to the medial aspect of the scapular spine. (LS, levator scapulae; Rm, rhomboid minor.)

**Fig 12:**
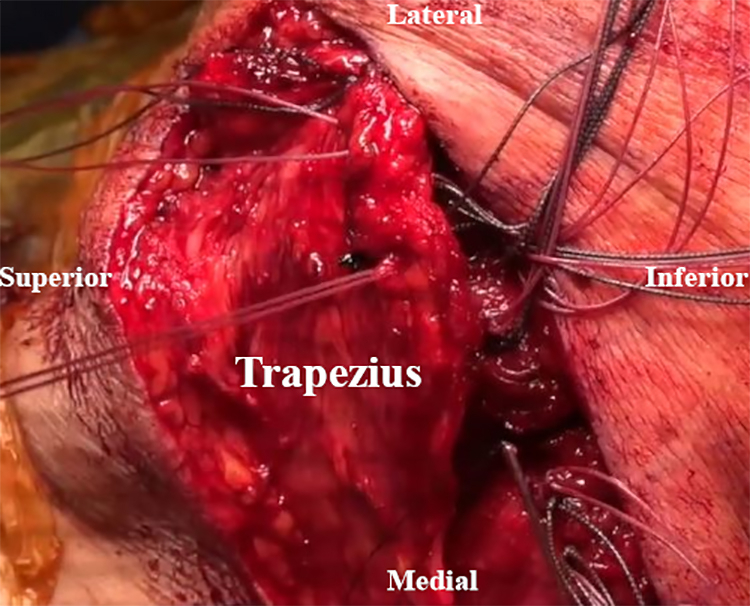
Intraoperative image of right scapula from posterior viewpoint. The paralyzed trapezius is repaired superficially to the reconstruction with sutures from the tendon transfer.

Prior to the tendon transfer, the anesthesiologist is asked to paralyzing agent the patient for tendon excursion and a smooth uninhibited transfer. The shoulder is placed in 70° of abduction to retract the scapula. The LS is transferred to the spine of the scapula slightly posterior to the posterior aspect of the acromion (Fig 9). The Rm is then transferred slightly medial to the attached LS along the scapular spine (Fig 10). The split bony attachment of the RM is fish-mouth and attached to the medial aspect of the scapular spine (Table 1). The proximal attachment is secured proximally on the medial scapular spine, and the distal attachment is secured to the distal-medial scapular spine (Fig 11). Throughout these steps, the dorsal scapular nerve is visualized and protected. The paralyzed trapezius flap is then placed over the reconstruction to avoid prominent knots. Sutures from the transfer are used to repair the paralyzed trapezius superficially to the reconstruction (Fig 12). Vancomycin powder is subsequently applied, and a Hemovac drain is placed.

**Table 1:** Pearls and Pitfalls

Step	Pearls	Pitfalls
Surgical approach	Semi-lateral decubitus positioning with appropriate and wide draping facilitates adequate exposure and efficient surgical technique.	Inadequate exposure increases the technical demand of the case and places the dorsal scapular nerve at risk of iatrogenic injury with inadequate visualization.
Tendon detachment and transfer preparation	Carefully placed tag sutures can be used for retraction, as well as later for tendon shuttling, with a 16- or 14-gauge Angiocath placed through bone tunnels.	Improper drill hole placement can risk scapular fracture or increase the difficulty of suture shuttling.
	The surgeon should make every effort to protect the dorsal scapular nerve.	Iatrogenic injury to the dorsal scapular nerve could result in palsy of the transferred tendons, therefore rendering the procedure futile.
Tendon transfer	Splitting the rhomboid major in a fish-mouth manner prior to transfer better approximates the native middle and lower trapezius line of excursion.	Failure to achieve paralysis of the patient prior to tendon transfer may result in inaccurate assessment of tendon excursion and improper tensioning, risking postoperative repair failure.
	Bone bridges should be used to provide biological healing of bone to bone.	Tendon transfer directly to bone may result in decreased biomechanical strength of the transfer.
Closure	The suture knots should be buried by repairing the trapezius over the top of the tendon transfer.	Failure to do so may result in knot prominence in the subcutaneous tissues with irritation.
Postoperative period	The postoperative protocol is time intensive and requires strict adherence to maximize functional outcomes. The importance of this should be emphasized during patient selection and education prior to surgery.	Failure to educate the patient prior to surgery can result in failure of the repair with a high potential for poor outcomes.
	Postoperative radiographic assessment of bony healing should be performed, with stepwise progression of activity as described in the “Postoperative Protocol” section as a general guide.	Progression of the patient too early may result in failure of repair.

### Postoperative Protocol

The drain is left intact until there is serosanguineous drainage of less than 30 mL/d. A custom shoulder abduction brace is used to allow 70° to 80° of abduction and 30° to 50° of external rotation. Assuming a smooth postoperative recovery, the patient may begin gentle active-assisted range of motion in the eighth postoperative week. At 12 weeks, the patient may begin full unrestricted range of motion. At 16 weeks, the patient may begin gentle progressive strengthening exercises. At 6 months, the patient may return to full unrestricted activity.

## Discussion

Trapezius paralysis is a rare and debilitating condition seen by orthopaedic surgeons. Patients with trapezius palsy often show a poor prognosis with nonoperative treatment and ultimately undergo surgical intervention for symptom relief.^3^ The Eden-Lange technique has historically been the gold standard for operative repair of trapezius paralysis. The original technique involves transfer of the LS to the lateral scapular spine, along with transfer of the Rm and RM to the infraspinatus fossa.^2^ Teboul et al.^4^ reported good to excellent outcomes in 4 of 7 patients who underwent the Eden-Lange transfer. In another study, Romero and Gerber^5^ reported good to excellent outcomes in 9 of 12 patients with the Eden-Lange technique. Myriad Eden-Lange modifications have been described in the previous literature. Bigliani et al.^6^ modified the technique by transferring the Rm to the supraspinous fossa, with excellent results in 13 of 22 patients and satisfactory results in 6 of 22. Recently, Elhassan and Wagner^7^ created a modified variant of the Eden-Lange technique in which the Rm and RM are transferred to the scapular spine medial to the LS transfer. Their study showed significant improvement with no drooping in 21 of 22 subjects at an average follow-up of 35 months postoperatively. Garcia et al.^8^ described a robotic transfer of the latissimus dorsi, in addition to mini-open LS and Rm transfers, for trapezius palsy, which in theory may shorten the duration of rehabilitation and avoid local soft-tissue adhesions.

The goal of our technique is to present surgeons with an efficient and reproducible step-by-step demonstration of a modified Eden-Lange transfer for patients with trapezius paralysis. Our technique varies from previously described modified Eden-Lange transfers in which a transverse incision is made at the RM to split it into 2 parts that are folded in a fish-mouth fashion prior to transfer. We believe these additional steps may provide better tendon excursion and improved biomechanics that better mimic the native middle and lower trapezius pull in patients with a prominent RM. The use of bone bridges may theoretically provide a higher load-to-failure rate than with tendon-to-bone healing. Another potential advantage of this technique is improved biomechanical function with medialization of the Rm to further mimic the middle trapezius fibers. Disadvantages of this technique include that it may be technically demanding and the surgical time may be lengthened. There is a risk of iatrogenic dorsal scapular nerve injury if dissection is not carefully performed. Patients should be carefully selected prior to undergoing this procedure owing to the extensive limitations with postoperative rehabilitation (Table 2). Further studies with long-term outcomes are suggested to evaluate whether this technique may produce superior outcomes to the original Eden-Lange transfer.

**Table 2:** Advantages and Disadvantages

Advantages
The modified Eden-Lange technique may better reproduce the natural biomechanical function of the middle trapezius fibers owing to medialization of the rhomboid minor.
Fish-mouthing the rhomboid major results in wider surface area coverage of the scapula, better mimicking the native function of the middle and inferior fibers of the trapezius.
Bone bridges provide biological bone-to-bone healing, theoretically resulting in a construct with higher load to failure than with tendon-to-bone healing.
With proper patient selection and education, this procedure may help improve pain and restore partial shoulder function.
Disadvantages
The procedure is technically demanding and time intensive.
An extensive postoperative rehabilitation protocol is required that may be unforgiving to noncompliant patients. Patient selection is critical.
There is a risk of iatrogenic injury to the dorsal scapular nerve, which innervates the transferred muscles; this could result in a failed procedure with worse outcomes than in the preoperative period, even with successful tendon transfer.
An inability to return to preinjury functionality or incomplete pain relief remains a risk because this technique was developed relatively recently, and patients should be counseled accordingly prior to surgical intervention.

## Supplementary Data

ICMJE author disclosure forms

Video 1 Intraoperative video of modified Eden-Lange triple tendon transfer in right scapula. The patient is placed in the semi-lateral decubitus position, with the surgeon operating through a single inverted L–shaped incision starting 1 cm proximal and 2 cm medial to the tip of the scapula.
